# Preventive effects of the butanol fraction of *Justicia procumbens* L. against dexamethasone-induced muscle atrophy in C2C12 myotubes

**DOI:** 10.1016/j.heliyon.2022.e11597

**Published:** 2022-11-22

**Authors:** Jae-Yong Kim, Hye Mi Kim, Ji Hoon Kim, Ju-Hee Lee, Kaixuan Zhang, Shuo Guo, Do Hyun Lee, Eun Mei Gao, Rak Ho Son, Seong-Min Kim, Chul Young Kim

**Affiliations:** a College of Pharmacy and Institute of Pharmaceutical Science and Technology, Hanyang University, Ansan, Gyeonggi-do, 15588, Republic of Korea; b R&D Center, Huons Co., Ltd., Ansan, Gyeonggi-do, 15588, Republic of Korea; c Medical Device Development Center, Daegu-Gyeongbuk Medical Innovation Foundation (DGMIF), Daegu, 41061, Republic of Korea

**Keywords:** *Justicia procumbens* L., Muscle atrophy, C2C12, Dexamethasone, Protein degradation

## Abstract

Skeletal muscle atrophy is associated with many diseases including cancer, inflammatory diseases, neuromuscular diseases, and acute critical illness. *Justicia procumbens* L. has been used as a herbal remedy, but the pharmacological effect of *J. procumbens* on muscle atrophy has not yet been reported. Herein, we investigate the anti-atrophic effect of the *n*-butanol fraction of *J. procumbens* (JPBuFr) on dexamethasone (DEX)-induced muscle atrophy in C2C12 myotubes. The myotubes diameter, MHC positive area, ROS production, and mitochondria contents were observed under a fluorescence microscope, and various proteins related to degradation or synthesis were analyzed by western blots. JPBuFr significantly attenuated a reduction of myotube diameter, mitochondrial content, ATP level, myosin heavy chain, and myogenin expression induced by DEX. Furthermore, co-treatment of DEX and JPBuFr not only increased phosphorylation of Akt, mTOR, and p70S6K proteins but also decreased reactive oxygen species production and expression of protein degradation factors (MuRF1, Atrogin-1, FoxO3a) compared to DEX treatment. These results suggest that JPBuFr may provide potential protective effects against muscle atrophy, giving it potential for the development of anti-atrophic health functional foods.

## Highlights

• Inhibitory activities of JPBuFr against DEX-induced muscle atrophy in C2C12 myotubes were investigated.
• JPBuFr reduces MuRF1, Atrogin-1, and FoxO3a expression in the DEX-induced muscle atrophy.
• JPBuFr promoted muscle protein synthesis via up-regulating the Akt/mTOR/p70S6K pathway in the DEX-induced muscle atrophy.
• JPBuFr effectively decreased ROS production as well as increased mitochondrial content and ATP levels in the DEX-induced muscle atrophy.

## 1 Introduction

Skeletal muscle is the most abundant and essential tissue in the human body, typically accounting for more than 40% and 30% of total body mass in men and women, respectively [1]. It plays many different critical functions, including maintaining posture and balance, respiratory mechanics, metabolism with whole-body homeostasis, insulin-stimulated glucose uptake and storage, and regeneration after injury [2]. Therefore, maintaining healthy muscles and therapies to treat and/or prevent muscle atrophy are necessary for protection against various diseases and healthy life.

Skeletal muscle atrophy is defined as a decrease in the size and mass of muscle tissue. It occurs when protein degradation exceeds protein synthesis [3]. There are many causes including a lack of physical activity, a sedentary lifestyle, starvation, aging, hormonal imbalance, severe injury, nerve damage, and many pathological conditions such as cancer, sepsis, obesity, diabetes, and immune disorders [4, 5]. In addition, muscle atrophy reduces the quality of life by causing exercise intolerance and difficulty in performing daily activities due to muscle weakness and fatigue [6]. Furthermore, excessive loss of muscle mass and function is associated with increased morbidity and mortality [7].

Many pathological conditions characterized by muscle atrophy are associated with elevated circulating glucocorticoid levels [8]. Dexamethasone (DEX), well known as a synthetic glucocorticoid, is commonly and effectively used as a therapeutic agent due to its powerful anti-inflammatory and anti-shock properties as well as its protection against autoimmune diseases [9]. Although DEX is useful, high dose or long-term consumption of DEX induces muscle atrophy through induction of proteolysis and inhibition of general protein synthesis [10]. It is well known that DEX increases the transcription of two muscle-specific ubiquitin E3 ligases, muscle atrophy F-box/atrogin-1 (MAFbx, also known as atrogin-1) and muscle ring finger protein 1 (MuRF-1), as well as other atrophy-related genes [11]. Upregulation of atrogin-1 and MuRF-1 is responsible for the increased protein degradation occurring during muscle atrophy. Furthermore, DEX induces muscle atrophy by inhibiting the phosphorylation of muscle protein synthesis factors including Akt, mTOR, p70S6K, and 4EBP1 in human skeletal muscle cells (HSkMCs) and mouse muscle cells (C2C12 cells) [12, 13].

*Justicia procumbens* L. (Acanthaceae), *Jwi-kko-ri-mang-cho* in Korean, is widely distributed in central and southern Korea, China, Japan, India, and Taiwan [14]. Previous studies have reported that *J. procumbens* is composed of various ingredients such as lignans, lignan glycosides, alkaloids, flavonoids, and triterpenes [15]. Lignans and lignan glycosides are major constituents of *J. procumbens*, which exhibits various pharmacological activities including anti-inflammatory, anti-tumor, anti-viral, anti-hepatitis, and anti-platelet aggregation [16]. In addition, the entire plant of *J. procumbens* has long been used as a herbal remedy for the treatment of fever, pain, cough, edema, jaundice, sore throat, urinary tract infection, and cancer [17, 18]. In addition, different types of cytotoxic activity have been reported in a partitioned extract of *J. procumbens* using water, petroleum ether, ethyl acetate, and *n*-butanol on human lung epithelial cell A549 [19]. Thus, the selection of relative fractions and treatment concentrations is important for an accurate evaluation of efficacy. In our preliminary study, we evaluated that the *n*-butanol fraction of *J. procumbens* significantly increased the reduction of myosin heavy chain *(*MHC) expression in C2C12 myotubes by DEX compared to the crude extracts of *J. procumbens* or its other fractions.

Although *J. procumbens* extract and its many ingredients have various pharmacological roles in the human body, its anti-skeletal muscle atrophy effect has not been investigated so far. Herein, we first report the preventive effects of the *n*-butanol fraction of *J. procumbens* (JPBuFr) on DEX-induced muscle atrophy and its molecular mechanisms in C2C12 myotubes.

## 2 Materials and methods

### 2.1 Plant materials

The plant materials of *J. procumbens* were collected from Anyang, Gyeonggi-do of the Republic of Korea in September 2020 and identified by one of the authors (CY Kim). A voucher was deposited at the Pharmacognosy Laboratory of the College of Pharmacy, Hanyang University (specimen no. HYUP-JP-001). An amount of 300 g of dried *J. procumbens* was extracted three times with 4 L of ethanol for 3 h at 70 °C, and the solvents were evaporated in vacuo at 40 °C, yielding the ethanol extract (8.80 g). The extract was suspended in water and then fractionated successively with equal volumes of *n*-hexane, ethyl acetate, and *n*-butanol.

### 2.2 Reagents

Dulbecco's Modified Eagle Medium (DMEM) 1640 medium, fetal bovine serum (FBS), and penicillin/streptomycin were purchased from Gibco Corporation (NY, USA). Dimethyl sulfoxide (DMSO), dexamethasone, and Hoechst 33342 were purchased from Sigma-Aldrich (MO, USA). A cell counting kit was purchased from Dojindo (Kumamoto, Japan). CM-H_2_DCFDA and MitoTracker Deep Red were purchased from Invitrogen (NY, USA). Blocking buffers and phosphate-buffered saline (PBS) were purchased from Biosesang (Seongnam, Korea). Ethanol, *n*-hexane, ethyl acetate, and *n*-butanol were purchased from Daejung Chemical (Siheung, Korea).

### 2.3 C2C12 cell culture and differentiation

Mouse myoblasts, C2C12 cells, were purchased from the American Type Culture Collection (ATCC, VA, USA). The cells were maintained in Dulbecco's Modified Eagle's Medium (DMEM) supplemented with 10% fetal bovine serum (FBS) and 1% penicillin/streptomycin at 37 °C in a humidified 5% CO_2_ atmosphere. To evaluate the anti-muscle atrophy effect, the C2C12 myoblasts were plated onto 12-well plates at a density of 5 × 10^4^ cells/well. Then, the cells were grown to 80–90% confluence in DMEM supplemented with 10% FBS at 37 °C for 48 h. Afterward, the medium was replaced with DMEM containing 2% Hores serum (HS) for 6 days to induce differentiation into myotubes, and it was replaced every 2 days.

### 2.4 Treatment of JPBuFr and dexamethasone

After 6 days of differentiation, the myotubes were subdivided into four groups as follows: (1) the control group, in which the cells were incubated in DMEM supplement 2% HS with 0.1% DMSO for 24 h; (2) the DEX-treated group, in which the cells were treated with 5 μM of DEX for 24 h; and (3) the DEX plus JPBuFr group, in which the cells were treated with DEX plus JPBuFr (10 and 20 μg/mL) for 24 h. Subsequently, all groups were harvested for the next experiments.

### 2.5 Cell viability measurements

The cytotoxicity of JPBuFr on C2C12 myotubes was evaluated using a cell counting kit-8 (CCK-8) cell viability assay kit (Dojindo, Kumamoto, Japan) according to the manufacturer's instructions. Briefly, differentiated C2C12 myotube cells (1 × 10^4^ cells/well) were cultured onto 48-well plates and then incubated with JPBuFr (5, 10, and 20 μg/ml) alone or co-treated with JPBuFr (5, 10, and 20 μg/ml) and Dex (5 μM) for 24 h at 37 °C in a humidified atmosphere of a 5% CO_2_ incubator. Subsequently, 20 μL of 2-(2-Methoxy-4-nitrophenyl)-3-(4-nitrophenyl)-5-(2,4-disulfophenyl)-2H-tetrazolium Sodium Salt (WST-8) solution was added to each well followed by a 4 h incubation. The absorbance was measured using an EnSpire Multimode Plate Reader at 450 nm (PerkinElmer, MA, USA).

### 2.6 Myosin heavy chain immunostaining

C2C12 myotubes were fixed with 4% paraformaldehyde for 10 min at room temperature and washed three times with phosphate-buffered saline (PBS). They were subsequently-permeabilized with 0.1% Triton X-100 for 20 min in PBS. After permeabilization, blocking was performed with 3% bovine serum albumin (BSA) for 1 h at room temperature. Then, the cells were incubated with MHC primary antibody (1:300, Santa Cruz, TX, USA) overnight at 4 °C. After washing three times with 0.1% PBST, the cells were incubated with a secondary antibody conjugated with Alexa Fluor 488 (1:500, Invitrogen, MA, USA) at 37 °C for 1 h. After washing three times with 0.1% PBST, the nuclei were counterstained with 10 μM of Hoechst 33342 (Sigma-Aldrich, MO, USA). The C2C12 myotube immunofluorescence was observed and captured using a fluorescence microscope (JuLI™ stage). The myotubes diameters were measured in a total 100 tubes from at least 5 different fields. For the MHC-positive area analysis, 5 randomly selected fields were counted from three independent experiments in each group. The myotubes diameters and MHC stained area were measured and analyzed using ImageJ software.

### 2.7 Western blot analysis

C2C12 myotubes were lysed with cold RIPA buffer (Invitrogen, MA, USA) containing protease inhibitor and phosphatase inhibitor cocktails on ice for 30 min. Whole-cell lysates were then centrifuged at 12,000×*g* for 20 min. The supernatants were transferred into new tubes, and the protein concentration of each sample was determined using the Pierce BCA Protein Assay Kit (Thermo Fisher Scientific, MA, USA). Equal amounts of protein (20 μg) from each well were loaded onto 8% SDS polyacrylamide gels and separated by electrophoresis. Afterward, it was transferred through electroblotting to the PVDF membranes (Merck, Darmstadt, Germany) for 1 h and blocked with 3% BSA solution for 2 h at room temperature. The membranes were incubated with different primary antibodies against MHC (1:1000, Santacruz, TX, USA); myogenin (1:1000, Santacruz, TX, USA); Akt (1:1000, Cell Signaling Technology, MA, USA); p-Akt (1:1000, Cell Signaling Technology, MA, USA); FoxO3a (1:500, Santacruz, TX, USA); mTOR (1:1000, Santacruz, TX, USA); p-mTOR (1:1000, Cell Signaling Technology, MA, USA); p70s6k (1:1000, Cell Signaling Technology, MA, USA); p-p70s6k (1:1000, Cell Signaling Technology, MA, USA); MuRF1 (1:1000, Santacruz, TX, USA); Atrogin-1 (1:1000, Santacruz, TX, USA); and GAPDH (1:10000, Santacruz, TX, USA) at 4 °C overnight. On the next day, the membranes were washed three times with 0.2% phosphate-buffered saline-Tween 20 (PBST) and incubated with the appropriate anti-mouse (1:5000, Santacruz, TX, USA) or anti-rabbit (1:5000, Santacruz, TX, USA) IgG HRP-linked antibodies for 2 h at room temperature. Subsequently, the membranes were washed three times with 0.2% PBST. Enhanced chemiluminescence (Thermo Fisher Scientific, MA, USA) was used to visualize the protein bands. Quantification of the protein band was analyzed using the Chemidoc imaging system (Biorad, CA, USA) and Image J software.

### 2.8 Measurement of intracellular ROS

The intracellular ROS content was measured using a fluorescent probe, CM-H_2_DCFDA. C2C12 cells were seeded in 24-well plates (3 × 10^4^/well) and fully differentiated into myotubes for 6 days. After differentiation, the myotubes were treated with 5 μM DEX in the presence or absence of JPBuFr (10 and 20 μg/ml, respectively) for 24 h. Subsequently, the myotubes were washed with PBS, and 10 μM of CM-H_2_DCFDA solution was added to each well and incubated for 15 min in the dark at room temperature. The cells were washed three times with a culture medium and then photographed using a fluorescence microscope (JuLI™ stage, Nano Entek, Seoul, Korea). CM-H2DCFDA positive cells were counted in at least 5 randomly selected fields from each well in three independent experiments. The fluorescence intensity was analyzed using Image J software.

### 2.9 Mitochondria staining

C2C12 cells were seeded in 24-well plates (3 × 10^4^/well) and fully differentiated into myotubes for 6 days. After differentiation, the myotubes were treated with 5 μM Dex in the presence or absence of JPBuFr (10 and 20 μg/ml, respectively) for 24 h. Subsequently, myotubes were stained with DMEM containing 500 nM Mito Tracker Deep Red for 30 min at 37 °C. Afterward, the cells were washed with PBS and fixed with 4% paraformaldehyde. After fixation, the cells were washed three times with PBS and then photographed using a fluorescence microscope (JuLI™ stage). Red fluorescence-positive cells were counted in at least 5 randomly selected fields from each well in three independent experiments. The fluorescence intensity was analyzed using Image J software.

### 2.10 Determination of the ATP level

ATP levels within the myotubes were measured using a luminescent ATP detection assay kit as described by the manufacturer (Cayman Chemical, MI, USA). Briefly, the myotubes were washed after treatment with cold phosphate buffer saline, and the myotubes were homogenized in the ATP detection sample buffer. The homogenization solution was centrifuged for 10 min at 13,000 *g*, and 10 μL of supernatant was transferred into each well of a 96-well white plate. Afterward, 100 μL of ATP reaction mix solution was added to each well and incubated at room temperature for 20 min. After the reaction, the ATP levels were measured using an EnSpire Multimode Plate Reader at 560 nm (PerkinElmer, MA, USA.)

### 2.11 Statistical analyses

All data are presented as the mean ± standard deviation (SD) for at least three independent experiments. Statistical significance was evaluated and determined by one-way analysis of variance (ANOVA) using GraphPad Prism 5.0 (GraphPad Software Inc, La Jolla, CA, USA), followed by Tukey's post hoc test. A *P*-value less than 0.05 was considered statistically significant.

## 3 Results

### 3.1 Effect of JPBuFr on viability in DEX-treated C2C12 myotubes

**Figure 1:**
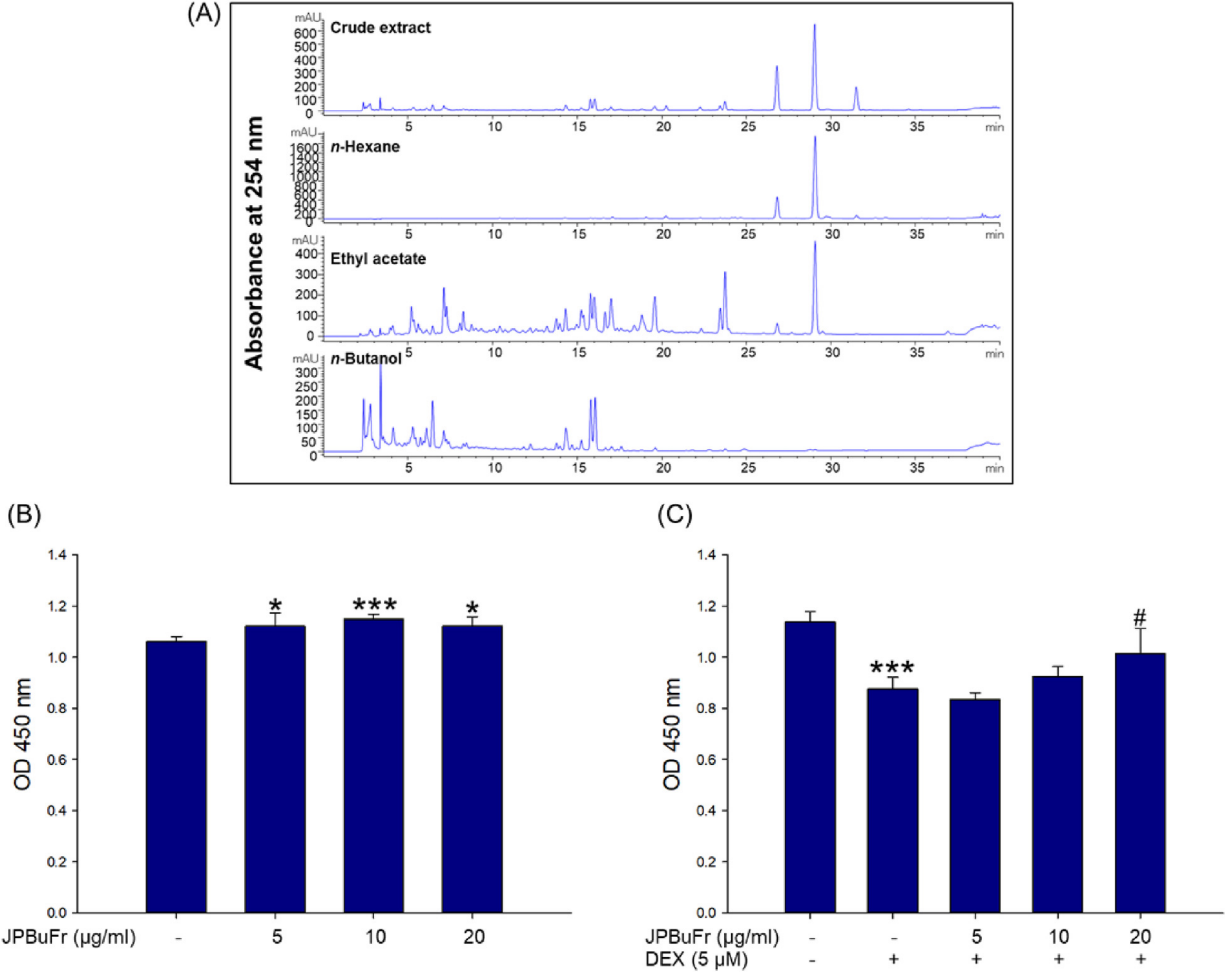
HPLC-UV/PDA chromatogram of *Justicia procumbens* L. and its subfractions and effects of JPBuFr and DEX on the viability of C2C12 myotubes. (A) The mobile phase consisted of acetonitrile (0.1 % formic acid, solvent A) and water (0.1 % formic acid, solvent B) in a gradient mode: 0 min, 20 % A; 20 min, 46 % A; 30 min, 55 % A; 35 min, 60 % A. The flow rate was 1 mL/min and the injection volume was 10 μL. The photodiode array (PDA) detector employed a UV spectrum over a range of 210–400 nm and the chromatogram of the effluents was recorded at 254 nm. (B) The cell viability was determined using the WST-8 assay. C2C12 myotubes were incubated with different concentrations of JPBuFr (5, 10, and 20 μg/ml) for 24 h. (C) C2C12 myotubes were treated with 5 μM DEX in the presence or absence of JPBuFr for 24 h. The values were presented as percentages of the control. These results are presented as means ± SD of three independent experiments. ∗*p* < 0.05, ∗∗∗*p* < 0.001 vs. control; ^#^*p* < 0.05 vs. DEX.

HPLC chromatogram of *Justicia procumbens* L extract and its *n*-hexane, ethyl acetate, and *n*-butanol fraction is shown in Figure 1A. We assessed the preventive effect of *J. procumbens* subfractions on the reduction of MHC protein expression by DEX in C2C12 myotube and found that the n-butanol fraction (JPBuFr) was the most effective (Supplementary Figure1). To determine the cytotoxicity and the appropriate concentration range for JPBuFr, fully differentiated C2C12 cells were treated at different concentrations (5, 10, and 20 μg/ml) for 24 h at 37 °C. In addition, to the protective effect of JPBuFr against DEX-induced cytotoxicity, the myotubes were incubated with various concentrations of the JPBuFr (5, 10, and 20 μg/ml) and 5 μM Dex for 24 h at 37 °C. The cell viability analysis showed that JPBuFr (5, 10, and 20 μg/ml) was not only non-toxic to C2C12 myotubes but also significantly increased compared to the control at all experimental concentrations (Figure 1B). DEX-treated myotubes exhibited a significant decrease in viability compared to the control (Figure 1C) whereas those co-treated with 5 μM DEX and JPBuFr (10 and 20 μg/ml) recovered cell viability (Figure 1C). In particular, co-treatment of JPBuFr (20 μg/ml) and 5 μM DEX significantly increased the viability compared with DEX-treated C2C12 myotube cells (Figure 1C). These results suggest that 10 and 20 μg/ml of JPBuFr were effective concentrations suitable for analyzing the effect on C2C12 myotubes against DEX-induced myotube atrophy.

### 3.2 Effect of JPBuFr on MHC expression and diameter in DEX-treated C2C12 myotubes

**Figure 2:**
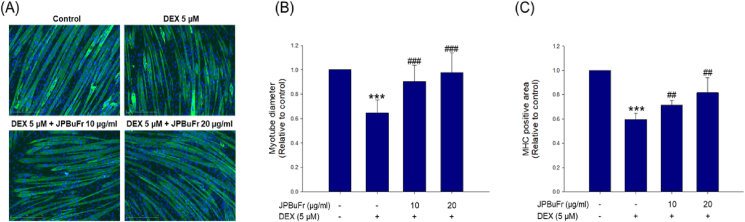
Effects of JPBuFr on DEX-induced atrophy in C2C12 myotubes. (A) The cells were fixed and immunostained with MHC (green) and DAPI (blue). Photographs were observed under a fluorescent microscope (scale bar = 250 μm). (B) Relative change in myotube diameters, and (C) MHC stained positive area were observed from randomly selected fields and were quantified using the image J program. These results are presented as means ± SD of three independent experiments: ∗∗∗*p* < 0.001 vs. control; ^##^*p* < 0.01, ^###^*p* < 0.001 vs. DEX.

The anti-atrophy effects of JPBuFr on C2C12 myotubes induced by DEX were evaluated by measuring the diameter and myosin heavy chain positive area. The DEX-treated group remarkably reduced the diameter of the myotube compared to the control whereas the DEX-treated with JPBuFr (10 and 20 μg/ml) showed significantly increased myotube diameters compared with the DEX-treated group in a concentration-dependent manner (Figure 2A and B). In particular, DEX-treated with 20 μg/ml of the JPBuFr groups exhibited almost the same level of diameter as the control group (Figure 2A and B). In addition, the DEX treated group crucially reduced the MHC positive area (0.60 ± 0.05) ability compared to the control (Figure 2C and D). However, the DEX-treated with JPBuFr (10 and 20 μg/ml) group remarkably recovered from the reduction of the MHC positive area (0.71 ± 0.04 and 0.82 ± 0.12, respectively) compared with DEX alone group (Figure 2C). These results demonstrate that JPBuFr markedly prevents DEX-induced muscle atrophy in C2C12 myotubes.

### 3.3 Effect of JPBuFr on the expression of muscle differentiation or degradation factors in DEX-treated C2C12 myotubes

**Figure 3:**
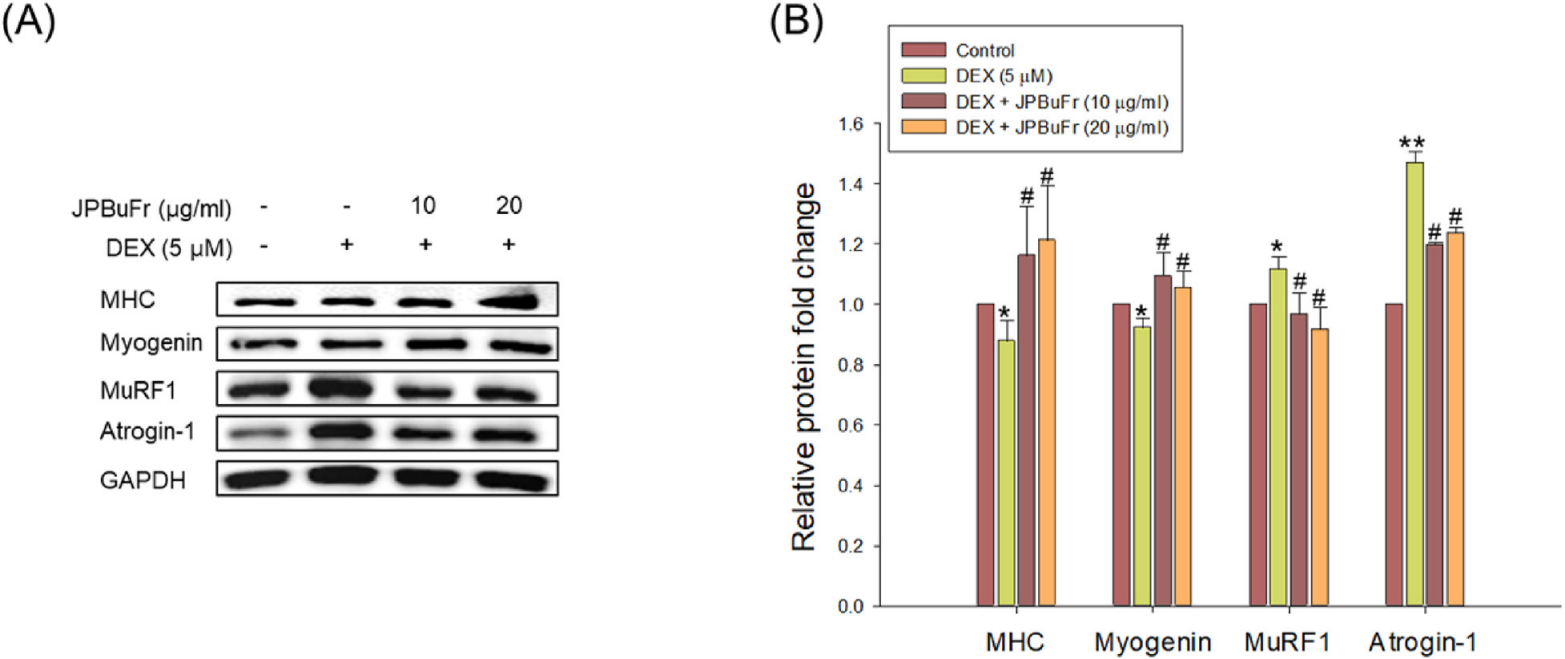
Effects of JPBuFr on the expression of muscle atrophy-related protein in DEX-induced atrophy in C2C12 myotubes. (A) Western blot of MHC, Myogenin, MuRF1, atrogin-1, and GAPDH proteins in cells treated with 5 μM DEX in the presence or absence of JPBuFr for 24 h. GAPDH was used as the loading control. (B) Quantitative analysis of MHC, Myogenin, MuRF1, and atrogin-1. The graph shows a quantitative representation of the levels of protein. These results are presented as the means ± SD of three independent experiments: ∗*p* < 0.05, ∗∗*p* < 0.01 vs. control; ^#^*p* < 0.05 vs. DEX.

To investigate the effect of JPBuFr on muscle differentiation or muscle atrophy in DEX-treated C2C12 myotubes, the expression levels of MHC, myogenin, MuRF1, and atrogin-1 were analyzed by Western blot. As a result, expression of MHC and myogenin was significantly decreased in 5 μM Dex-treated myotubes, while MuRF1 and atrogin-1 levels were robustly increased (Figure 3A and B). In contrast, JPBuFr (10 or 20 μg/ml) treatment significantly increased the expression of MHC and myogenin compared to that in DEX-treated C2C12 myotubes. In addition, JPBuFr (10 and 20 μg/ml) treatment also significantly reduced the expression of MuRF1 and atrogin-1 compared to the DEX-treated C2C12 myotubes (Figure 3A and B). These results indicate that JPBuFr prevents DEX-induced atrophy in C2C12 myotubes by restoring the expression of MHC and myogenin as well as suppressing the expression of MuRF1 and atrogin-1.

### 3.4 Effect of JPBuFr on the expression of p-Akt, p-mTOR, p-p70S6K, and FoxO3a in Dex-treated C2C12 myotubes

**Figure 4:**
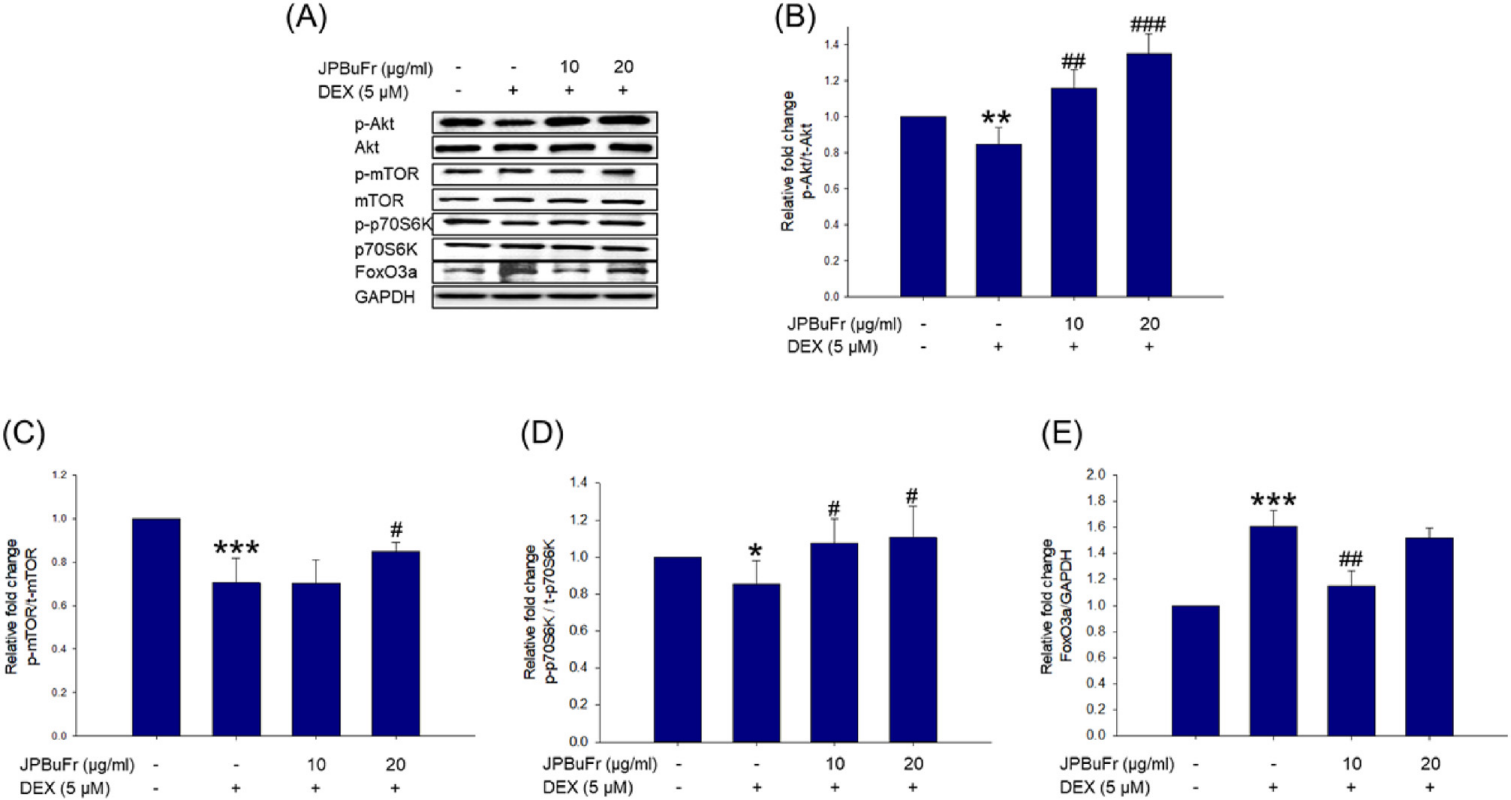
Effects of JPBuFr on the Akt/mTOR/p70S6K and Akt/FoxO3a pathway in DEX-induced atrophy in C2C12 myotubes. (A) Western blot of p-AKT, AKT, p-mTOR, mTOR, p-p70S6K, p70S6K, and FoxO3a proteins in the C2C12 myotubes treated with 5 μM DEX in the presence or absence of JPBuFr for 24 h. (B) Quantitative analysis of the p-Akt/Akt. (C) Quantitative analysis of the p-mTOR/mTOR. (D) Quantitative analysis of the p-p70S6K/p70S6K. (E) Quantitative analysis of the FoxO3a. The graph shows a quantitative representation of the levels of protein. These results are presented as the means ± SD of three independent experiments: ∗*p* < 0.05, ∗∗*p* < 0.01, ∗∗∗*p* < 0.001 vs. control; ^#^*p* < 0.05, ^##^*p* < 0.01, ^###^*p* < 0.001 vs. DEX.

To investigate the mechanism of action of JPBuFr on the preventive activity of muscle atrophy, Akt, mTOR, p70s6k, and FOXO3a proteins, which are signaling pathway factors related to protein synthesis and degradation, were analyzed by Western blot in DEX and/or JPBuFr treated C2C12 myotubes. When C2C12 myotubes were treated with 5 *μ*M DEX for 24 h, the phosphorylation of Akt, mTOR, and p70S6K significantly decreased compared to the control (Figure 4A, B, C, and D). JPBuFr treatment restores phosphorylation of Akt and p70S6K levels significantly in DEX-treated C2C12 myotubes. Surprisingly, the relative levels of p-Akt and p-p70S6k were higher than that of the control group at both concentrations (Figure 4A, B, and D). In addition, the phosphorylation of mTOR did not differ at 10 μg/ml of JPBuFr but was significantly increased at 20 μg/ml (Figure 4A and C) compared to the DEX-treated group. On the other hand, when DEX was treated, the expression of Foxo3a was significantly increased compared to the control, but the co-treated with JPBuFr (at 10 μg/ml) group showed a significantly decreased expression of Foxo3a in C2C12 myotubes (Figure 4A and E). These results indicate that JPBuFr had protection activity against atrophy through the up-regulation of the Akt/mTOR/p70S6K signaling pathway and down-regulation of the Akt/FoxO3a signaling pathway in DEX-treated C2C12 myotube cells.

### 3.5 Effect of JPBuFr on reactive oxygen species production in DEX-treated C2C12 myotubes

**Figure 5:**
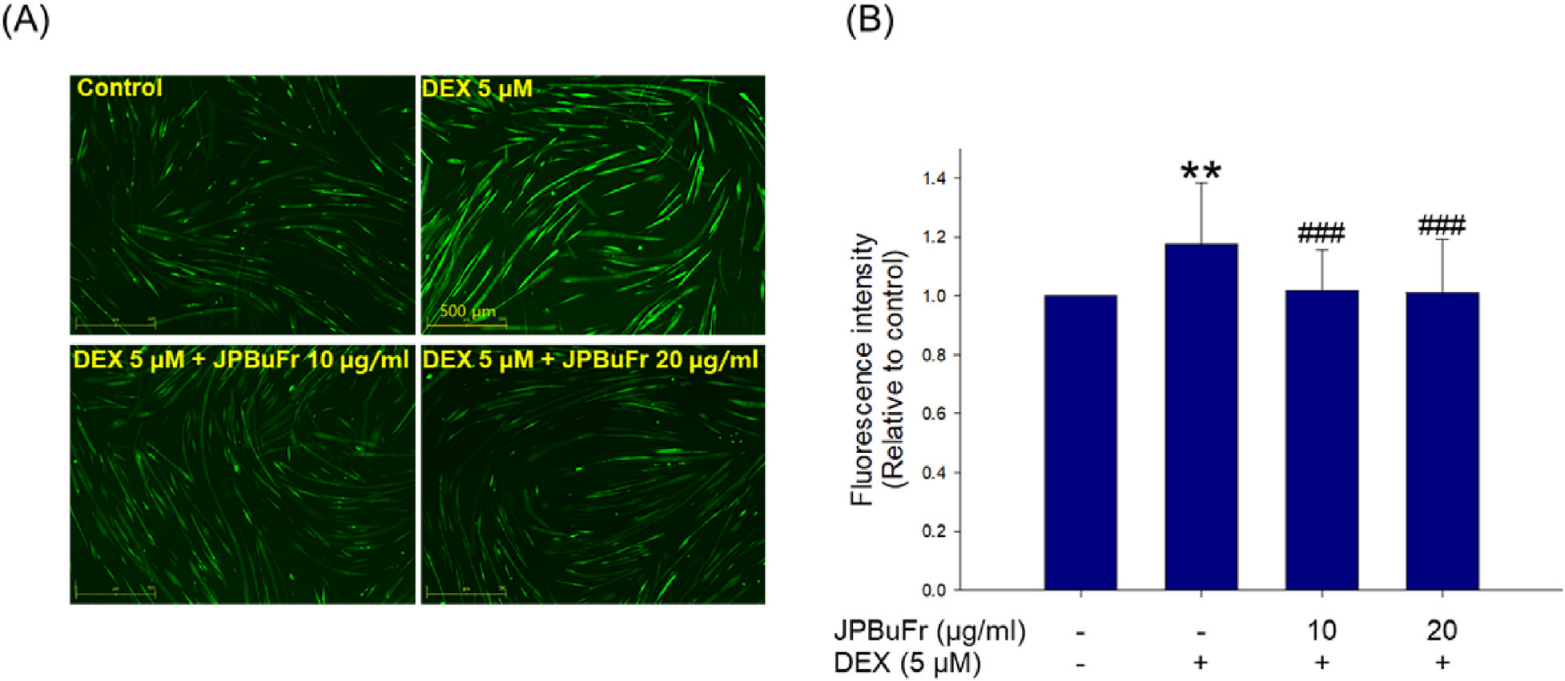
Effects of JPBuFr on ROS production in DEX-induced atrophy in C2C12 myotubes. (A) The production of cellular ROS analysis using a 2,7-dichlorodihydrofluorescein diacetate (DCFH-DA) dye and observed by a fluorescence microscope (scale bar = 500 μm). (B) The graph shows the quantification of the CM-H_2_DCFDA stained area using the Image J software program. These results are presented as means ± SD of three independent experiments: ∗∗*p* < 0.01 vs. control; ^###^*p* < 0.001 vs. DEX.

Fluorescent probe CM-H_2_DCFDA staining was used to measure the production of reactive oxygen species. ROS levels of DEX-treated C2C12 myotube cells were significantly increased compared with the control (Figure 5A and B). However, co-treatment of DEX with JPBuFr (10 and 20 μg/ml) remarkably reduced ROS production compared to the DEX-treated C2C12 myotubes and even showed similar results to the control group (Figure 5A and B).

### 3.6 Effect of JPBuFr on mitochondria loss and ATP production ability in DEX-treated C2C12 myotubes

**Figure 6:**
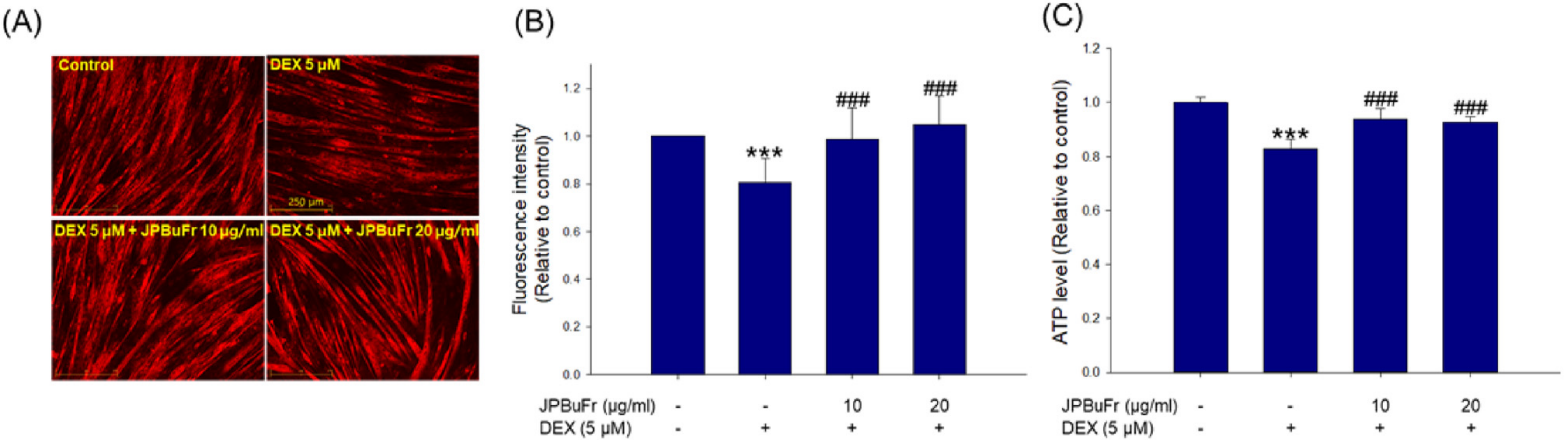
Effects of JPBuFr on the mitochondrial content and ATP levels in DEX-induced atrophy in C2C12 myotubes. (A) The mitochondrial contents were determined by MitoTracker Red staining and observed by a fluorescence microscope (scale bar = 250 μm). (B) The graph shows the quantification of the MitoTracker Deep Red stained area by the Image J software program. (C) ATP production in the C2C12 myotubes. These results are presented as means ± SD of three independent experiments: ∗∗∗*p* < 0.001 vs. control; ^###^*p* < 0.001 vs. DEX.

Quantitative and qualitative analysis of mitochondria in C2C12 myotubes was performed by MitoTracker Deep Red staining and measurement of the ATP level. Treatment of DEX caused a clear decrease in mitochondrial contents and ATP levels in C2C12 myotubes compared to the control group (Figure 6A, B, and C). On the other hand, JPBuFr (10 and 20 μg/ml) treated C2C12 myotubes significantly prevented the decrease in mitochondrial content and ATP production capacity caused by DEX, respectively (Figure 6A, B, and C). Surprisingly, the mitochondrial content of the C2C12 myotube co-treated with 20 μg/ml of JPBuFr and DEX was higher than that of the control group.

## 4 Discussion

Muscle atrophy occurs in a variety of physiological and pathological conditions, including inactivity or muscle disuse, fasting, and various diseases such as sepsis, cachexia, cancer, diabetes, and many chronic diseases as well as causing an increase in morbidity and mortality. Hence, maintaining healthy muscle mass is necessary for achieving a healthy life.

Various natural plants and their compounds are being studied as candidates for drugs for various diseases due to their advantages of having few side effects, great activity, and structural diversity [20]. Many natural compounds (polyphenol, flavonoids, alkaloids, triterpenoids, and others) have been reported for their preventive effects for muscle atrophy and their potential as therapeutic agents [21]. To date, many studies on the biological activity of *J. procumbens* have been reported, but there has been no report on the effect and molecular mechanism of *J. procumbens* on muscle atrophy. Here, we first report the preventive activity of muscle atrophy and the mechanism of action of *J. procumbens* against DEX-induced C2C12 myotube atrophy. Before conducting our experiments, we confirmed that the *n*-butanol fraction of *J. procumbens* (JPBuFr) significantly restored the DEX-induced reduction of MHC protein levels in C2C12 myotubes compared to the whole extract of *J. procumbens* as well as other fractions (Supplementary Figure1). Based on these results, all further experiments were performed following treatment with JPBuFr.

A previous study showed that C2C12 viability was significantly reduced when treated with 10 μM DEX for 24 h, and the cytotoxicity was severely inhibited by Myricanol, isolated from the bark of *M*. *rubra* [22]. However, the effect of JPBuFr on DEX-induced cytotoxicity in C2C12 myotubes has not been reported. In this study, cytotoxicity was measured by the WST-8 assay, which is the most commonly used to evaluate cell viability and cytotoxicity [23]. Our current results showed that JPBuFr (5, 10, and 20 μg/ml) had no cytotoxicity in C2C12 myotubes (Figure 1B), and in particular, 10 and 20 μg/ml of JPBuFr inhibited the cytotoxicity caused by 5 μM Dex (Figure 1C). These results suggest that 10 and 20 μg/ml of JPBuFr effectively recovered the cytotoxicity to C2C12 myotubes by DEX, thereby inhibiting skeletal muscle cell damage.

Many previous studies have shown that DEX reduces the diameter and MHC expression of C2C12 myotubes, which are known as representative phenotype modifiers of muscle atrophy [24, 25]. In the present study, we analyzed the effect of JPBuFr on DEX-induced atrophy in C2C12 myotubes by immunostaining with MHC, a representative marker of myotube differentiation [26]. As shown in Figure 2, the diameter and MHC expression of C2C12 myotubes decreased following DEX treatment but were significantly recovered by JPBuFr treatment (at 10 and 20 μg/ml). Therefore, JPBuFr can prevent muscle atrophy by effectively restoring the inhibition of C2C12 myotube differentiation by DEX.

Myogenin (Myog) and MHC are the major developmental regulators of skeletal muscle formation and differentiation [27, 28]. Many in vitro studies have reported that an increase in skeletal muscle differentiation is promoted by up-regulation of Myog and MHC expression [29, 30]. On the other hand, muscle RING finger 1 (MuRF1) and muscle atrophy F-box (MAFbx)/atrogin-1 are the two most well-known muscle-specific E3 ubiquitin ligases and are key markers of muscle atrophy [11]. MuRF-1 induces the degradation of muscle structural proteins including myosin heavy chain, actin, myosin-binding protein C, and troponin I [31, 32]. Atrogin-1 appears at an early stage before muscle loss is detected and promotes the degradation of eukaryotic translation initiation factor 3 (eIF3), a protein translation initiation factor [33, 34]. Several studies have revealed that DEX induces muscle atrophy in C2C12 myotubes by inhibiting Myog and MHC expression and increasing the expression of MuRF1 and atrogin-1. These characteristics are alleviated by natural substances such as mirycanol, fucoxantine, and matrine [22, 24, 35]. Although some natural substances have anti-atrophic effects in DEX-treated C2C12 myotubes via up-regulation of Myog and MHC and down-regulation of MuRF1 and atrogin-1, the effect of JPBuFr has not been elucidated. In the present study, Myog and MHC expression was remarkably reduced by DEX treatment compared to the control (Figure 3A and B). On the other hand, MuRF1 and atrogin-1 were significantly higher than that of the control (Figure 3A and B). However, when JPBuFr and DEX were co-treated, not only the increase in myogenesis factors (MHC and Myog) but also a decrease in the protein degradation factors (MuRF1 and atrogin-1) were found (Figure 3A and B). These results suggest that JPBuFr prevented DEX-induced atrophy in C2C12 myotubes by promoting inhibition of muscle protein degradation and increased synthesis.

There are several signaling pathways involved in skeletal muscle hypertrophy and atrophy, including regulation of protein synthesis and degradation [36]. The Akt/mTOR/p70S6K signaling pathway plays a crucial role in the process of protein synthesis. The serine/threonine kinase Akt is an important regulator of various cellular functions including survival, growth, differentiation, metabolism, and migration [37]. Akt activation led to an increase in mTOR phosphorylation and induced up-regulation of protein synthesis in muscles [38]. In addition, mTOR is involved in multiple cellular responses including cell growth, proliferation, and survival [39]. Another pathway is p70S6K, a mitogen-activated Ser/Thr protein kinase required for cell growth and G1 cell cycle progression, which is activated in signaling pathways involving mTOR [40]. Therefore, it is considered that up-regulation of the AKT/mTOR/p70S6K signaling pathway could prevent the development of muscle atrophy by increasing protein synthesis.

FoxO3a regulates the ubiquitin-proteasome system during atrophy and accelerates protein degradation [41]. FoxO3a translocates to the nucleus as a transcription factor upon dephosphorylation, eventually upregulating E3 ubiquitin ligases such as MuRF1 and atrogin-1 [24]. In addition, FoxO3a is phosphorylated by Akt at three residues: threonine32, serine253, and serine315. This causes it to be excluded from the nucleus as well as resulting in FoxO3a inhibition [42]. Thus, the AKT/FoxO3a signaling pathway is considered a potential target of muscle atrophy. It has been shown in several C2C12 myotube and mouse models that DEX significantly inhibits the Akt/mTOR/p70S6K signaling pathway and increases FoxO3a expression, leading to muscle atrophy [13, 24, 25]. For this reason, we investigated the mechanism of action of JPBuFr on inhibition of DEX-induced muscle atrophy in C2C12 myotubes by analyzing the phosphorylation of Akt, mTOR, and p70S6K as well as the expression of the FoxO3a protein assessed by Western blot. In our results, treatment of 5 *μ*M DEX significantly decreased the phosphorylation of Akt, mTOR, and p70S6K compared to the control (Figure 4A–D). However, the relative levels of phosphorylation of Akt and p70S6K were significantly increased by JPBuFr treatment (at 10 and 20 μg/ml) compared to the DEX-treated group (Figure 4A, B, and D). The mTOR phosphorylation was also markedly improved by JPBuFr (at 20 μg/ml) compared to the DEX-treated group (Figure 4A and C). Moreover, 10 μg/ml of JPBuFr effectively reduced the up-regulation of Foxo3a by DEX (Figure 4A and E). Therefore, these results suggest that JPBuFr upregulates the anabolism and downregulates the catabolism of muscle-specific proteins.

Reactive oxygen species alter cellular function by destroying and oxidizing proteins, lipids, and DNA [43]. ROS production stimulates protein degradation in a muscle by increasing the two muscle-specific ubiquitin E3 ligases, atrogin-1 and MuRF1. Overproduction of ROS is closely associated with skeletal muscle atrophy [44, 45]. Therefore, materials that inhibit ROS production could potentially be used in therapeutic intervention for skeletal muscle atrophy. It has been reported that DEX induced excessive generation of ROS in osteoblast-like cells (MC3T3-E1) and human umbilical vein endothelial cells (HUVEC) [46, 47]. In addition, recent studies have shown that the increase of ROS formation in atrophy-induced C2C12 myotubes by DEX was significantly reduced by co-treatment of some natural products such as quercetin, morin, and *Valeriana fauriei* [48, 49, 50]. However, the preventive activity of JPBuFr on DEX-induced ROS formation in C2C12 myotubes is not yet clear. Here, to determine the efficacy of BuFr on ROS production, we used fluorescent probe CM-H_2_DCFDA staining and subsequently measured the fluorescence intensity values using a fluorescence microscope [51]. In the present study, ROS production was significantly increased in DEX-treated C2C12 myotubes (Figure 5A and B), which is in good agreement with the previously published experimental results. The overproduction of ROS by DEX was reduced to a level similar to that of the control due to the JPBuFr treatment (Figure 5A and B). These findings suggest that JPBuFr effectively prevented ROS production and eventually inhibited muscle atrophy by DEX.

Mitochondrial dysfunction is implicated in many human diseases including cancer, neurodegenerative disorders, and metabolic syndrome [52]. It also promotes skeletal muscle wasting and appears in various models of muscle atrophy, including disuse, diabetes, and aging [53, 54, 55, 56]. It is well known that mitochondrial content and ATP production capacity play an important role in maintaining normal mitochondrial function and muscle function [57, 58]. A previous study reported that DEX induces muscle atrophy by promoting mitochondrial dysfunction through decreased ATP production as well as reduced mitochondrial content in C2C12 myotubes compared to the native state [59]. These results suggest quantitative and qualitative improvement of mitochondria can be used as a means to prevent muscle atrophy. In the present study, we evaluated the effect of JPBuFr on the inhibition of mitochondrial dysfunction in atrophy-induced C2C12 myotubes by DEX. Mitochondrial content and ATP levels were found to be significantly reduced compared to the control; however, these reductions were restored to a level similar to that of the control by JPBuFr treatment (Figure 6A, B, and C). These results indicate that JPBuFr can protect against muscle atrophy by inhibiting mitochondrial dysfunction in DEX-treated C2C12 myotube cells.

## 5 Conclusion

JPBuFr has a protective effect against DEX-induced atrophy in C2C12 myotubes via inhibition of protein degradation, reduction of ROS production, prevention of mitochondria dysfunction, and improvement of protein synthesis, suggesting that JPBuFr is a potential supplement for the prevention of muscle atrophy. However, further studies investigating other possible anti-atrophy-related mechanisms and in vivo efficacies for JPBuFr are required.

## Declarations

### Author contribution statement

Jae-Yong Kim: Conceived and designed the experiments; Performed the experiments; Analyzed and interpreted the data; Wrote the paper.

Hye Mi Kim: Performed the experiments; Analyzed and interpreted the data; Wrote the paper.

Ji Hoon Kim: Performed the experiments.

Ju-Hee Lee, Kaixuan Zhang, Shuo Guo and Do Hyun Lee: Analyzed and interpreted the data.

Eun Mei Gao, Rak Ho Son and Seong-Min Kim: Contributed reagents, materials, analysis tools or data.

Chul Young Kim: Conceived and designed the experiments.

### Funding statement

Chul Young Kim was supported by 10.13039/501100001321National Research Foundation (Republic of Korea) [NRF-2020R1A2C1009455 & NRF-2020R1A6A1A0-3042854].

Jae-Yong Kim was supported by 10.13039/501100001321National Research Foundation (Republic of Korea) [NRF-2020R1I1A1A01069216].

### Data availability statement

Data included in article/supp. material/referenced in article.

### Declaration of interest's statement

The authors declare no conflict of interest.

### Additional information

No additional information is available for this paper.

## Appendix A Supplementary data

supplementary data_HELIYON-D-22-18142 (HLY e11597)_V2.docx supplementary data_HELIYON-D-22-18142 (HLY e11597)_V2.docx
