# Supplementary material for: Preventive effects of the butanol fraction of Justicia procumbens L. against dexamethasone-induced muscle atrophy in C2C12 myotubes
Source: Heliyon. 2022 Nov 22;8(11):e11597. doi: 10.1016/j.heliyon.2022.e11597 (PMC9713280; doi:10.1016/j.heliyon.2022.e11597)
Supplement: supplementary data_HELIYON-D-22-18142 (HLY e11597)_V2.docx [file mmc1.docx]

Preventive effects of the butanol fraction of *Justicia procumbens* L. against dexamethasone-induced muscle atrophy in C2C12 myotubes

Jae-Yong Kim^1,†^, Hye Mi Kim^1,†^, Ji Hoon Kim^1^, Ju-hee Lee^1^, Kaixuan Zhang^1^, Shuo Guo^1^, Do-hyun Lee^1^, Eun Mei Gao^1^, Rak Ho Son^1,2^, Seong-Min Kim^3^ and Chul Young Kim^1,*^

^1^ College of Pharmacy and Institute of Pharmaceutical Science and Technology, Hanyang University, Ansan, Gyeonggi-do, 15588, Republic of Korea

^2^ R&D Center, Huons Co., Ltd., Ansan, Gyeonggi-do, 15588, Korea

^3^ Medical Device Development Center, Daegu-Gyeongbuk Medical Innovation Foundation (DGMIF), Daegu, 41061, Republic of Korea

*Corresponding authors (Tel: +82 31 400 5809; Fax: +82 31 400 5958; E-mail: [chulykim@hanyang.ac.kr](mailto:chulykim@hanyang.ac.kr).)

†These authors contributed equally to this work.

**Fig. S1**. Effect of *Justicia procumbens* L. extract and its subfractions on MHC expression in DEX-treated C2C12 myotubes.

*
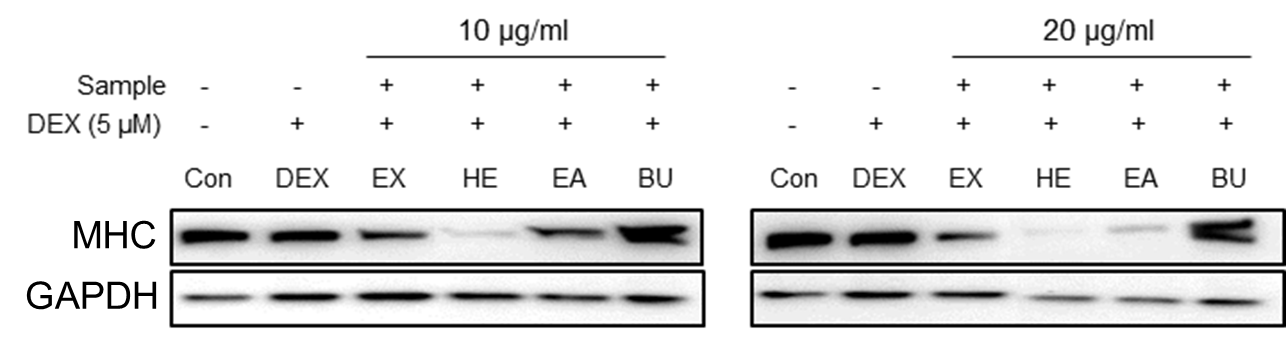
*

**Fig. S1. Effects of *Justicia procumbens* L extract and its subfractions on the expression of MHC protein in C2C12 myotubes.** Western blot of MHC in C2C12 myotubes treated with 5 μM dexamethasone in the presence or absence of JPL extract or its sub-fractions for 24 h. GAPDH was used as a loading control. Con, Control; DEX, Dexamethasone; EX, JPL extract; HE, *n*-hexane; EA, Eethyl acetate; BU, *n*-butanol.
